# Polymicrobial Infection (Gram-Positive and Gram-Negative) Exacerbates Systemic Inflammatory Response Syndrome in a Conscious Swine Extremity Trauma Model

**DOI:** 10.3390/pathophysiology32040059

**Published:** 2025-11-04

**Authors:** Catharina C. Gaeth, Travis R. Madaris, Jamila M. Duarte, Amber M. Powers, Christina M. Sandoval, Stefanie M. Shiels, Randolph Stone

**Affiliations:** Combat Wound Care, United States Army Institute of Surgical Research, 3698 Chambers Pass, Fort Sam Houston, TX 78234-6315, USA

**Keywords:** preclinical trauma models, SIRS, extremity trauma, prolonged casualty care

## Abstract

**Background/Objectives**: Extremity trauma represents a significant proportion of battlefield injuries and is prevalent in polytraumatized patients from accidents. Delayed antibiotic treatment and surgical intervention can lead to wound infections, contributing to preventable mortality. This preliminary study aimed to develop a conscious swine model of complex extremity trauma that induces systemic inflammatory response syndrome (SIRS). **Methods**: All surgical procedures were conducted under anesthesia with sufficient analgesia. All swine were instrumented with a telemetry device and catheters at least 3 days prior to any injury. In phase 1 of model development, a complex extremity injury was performed that consisted of skin and muscle loss, bone defect, severe hemorrhage, and 2 h tourniquet application. In phase 2, multi-drug resistant Gram-positive and Gram-negative bacteria were inoculated topically at the injury site to exacerbate pathophysiological changes towards SIRS. Post-injury, conscious animals were assessed a minimum of twice daily, including pain assessment, neurological response, and vital signs. Blood samples were collected for microbiological testing, complete blood cell counts, and biochemical analysis. **Results**: After establishing SIRS criteria for Sinclair swine, we developed a model of severe extremity trauma leading to SIRS. During phase 1, resuscitative fluids were reduced and discontinued, with animals surviving 24 h and maintaining SIRS for up to 4 h post-recovery. Phase 2 showed that Gram-negative and Gram-positive pathogens can exacerbate and prolong SIRS. After 72 h, localized infection at the injury site was observed in all animals. **Conclusions**: We established a new swine model of complex extremity trauma with SIRS. Our model is consistent, reproducible, and relevant to prolonged care scenarios, providing a platform for future research into the evaluation of preventative and therapeutic strategies.

## 1. Introduction

Prior military conflicts have shown that most combat injuries are caused by mechanisms such as explosive devices, and gunshot wounds. They often result in complex injuries with extensive wounds as well as severe damage to multiple organs, limbs, and major blood vessels that require urgent medical care. While body armor effectively protects the head, chest, and abdomen, the extremities remain vulnerable, making them particularly susceptible to serious injury and related complications such as blood loss and infection [1]. Extremity trauma, with injuries such as open fractures and soft tissue damage, accounted for 51.9% of combat wounds in the Iraq and Afghanistan conflicts [2]. Within this population, the incidence of infection reached as high as 75% in severe open fractures such as Gustilo Anderson type IIIC [3]. The risk of infection is a major challenge in the treatment of both military and civilian trauma [4,5,6]. Current battlefield medical care approaches are geared toward stopping bleeding, providing fluids, and temporary wound stabilization and are highly dependent on quick transport to facilities providing surgical care [7,8]. Delays in evacuation are expected in future conflicts, which may lead to an increase in complications and mortality.

During severe trauma, illness, or infection, a non-specific, system-wide response, known as systemic inflammatory response syndrome (SIRS), may develop [9,10]. SIRS describes the immediate body reaction, which consists of pro- and anti-inflammatory cascades at the cellular level for defense purposes of the body [11]. Early, fast diagnosis and treatment are crucial to prevent aggravation and systemic spread of infection which significantly increases both morbidity and mortality [4,9,10,12,13]. Scoring systems, like the SIRS criteria, which consist of heart rate (HR), respiratory rate (RR), temperature, and white blood cell count (WBC), assist medical care providers in identifying when a SIRS state develops [10,12,13,14,15].

Preclinical animal models of extremity trauma are useful tools to better understand the pathophysiology of trauma, optimize treatment protocols, and develop new technologies. Yet, existing extremity trauma models have limitations; often using small animals or investigating polytraumatized animals with additional organ damage, such as lung contusion or liver laceration [15,16,17]. This underscores the need for a specific extremity trauma model that focuses exclusively on severe extremity trauma and its complications, as well as the physiological response in terms of SIRS.

The aim of this study was to establish an infection model of complex extremity trauma that resulted in a SIRS state. Swine have anatomical and physiological similarities to humans and have been used to mimic other clinically relevant traumas [18,19]. The animal model was developed in 2 phases. Phase 1 optimized the extremity injury, hemorrhage, and resuscitation procedures to acutely induce SIRS. Phase 2 evaluated if extending the post-injury monitoring period for 72 h and inoculating the wound with bacteria would result in a sustained SIRS state. In the future, this model will help to study the effectiveness of decision support tools, diagnostic devices, and therapeutic interventions over a prolonged post-injury period for infected extremity injuries [19].

## 2. Materials and Methods

### 2.1. Animals

Research was conducted in compliance with Animal Welfare Act, implementing Animal Welfare regulations and the principles of the Guide for the Care and Use of Laboratory Animals. The Institutional Animal Care and Use Committee approved all research conducted in this study. The facility where this research was conducted is fully accredited by the AAALAC International. For this study, 26 adult, castrated, male Sinclair miniature swine (Sinclair Bio Resources, Auxvasse, MO, USA), aged 17.1 ± 2.4 months with an average weight of 40.8 ± 3.9 kg were used (Phase 1: N = 11; Phase 2: N = 11). Sinclair miniature swine reach adult physiological maturity while maintaining a relatively small body size. Furthermore, based on our prior experience with different breeds of swine, we have found that Sinclair swine have the most suitable anatomical features for the standardized application of tourniquets for translational research. Four animals only provided baseline data for the development of the SIRS criteria but were not included in any other analysis due to either not surviving the injury procedure or not receiving all the same procedures after injury.

The swine were acclimatized for one week. Subsequently, each animal received positive reinforcement behavior training which allowed routine blood draws while the animals were conscious in their pens [20]. Experimental details are reported following the ARRIVE (Animal Research: Reporting of In Vivo Experiments) guidelines.

### 2.2. Analgesics and Anesthesia

Prior to surgical procedures, animals had unrestricted access to food and water. Animals were fasted overnight prior to each anesthetic event. All animals received preemptive analgesia (buprenorphine extended release (0.1–0.24 mg/kg) providing up to 72 h of coverage prior to each surgical procedure and were monitored for pain and distress following recovery. Breakthrough pain was treated with additional analgesia depending on daily pain assessments carried out by personnel experienced in identifying signs of pain in swine.

On the day of surgery, for reduction in vagal bradycardia, the neck was injected with glycopyrrolate [0.01 mg/kg, via intramuscular (IM)]. Anesthesia was induced with ketamine (10–25 mg/kg, IM) or tiletamine-zolazepam (4–6 mg/kg, IM). Initial isoflurane was maintained at 3–5% (*v*/*v*) in oxygen via a face mask. Animals were intubated. The ventilator settings were set to the following: tidal volume (8–12 mL/kg), peak pressure [20 cm H_2_O and 8–20 breaths per minute (brpm)], end-tidal PCO_2_ (40 ± 5 mmHg). Throughout the procedures, 1–3% isoflurane in oxygen was maintained.

### 2.3. Surgical Instrumentation

At least 3 days before the experimental injury, animals were anesthetized. The carotid artery and jugular vein were catheterized. The catheters were tunneled to exit out the dorsum of the neck for conscious blood collection. The right femoral artery was used to implant a DSI (Data Sciences International, New Brighton, MN, USA) wireless telemetry device to continuously collect data for HR, RR, temperature, blood pressure, and activity. The DSI implant was sutured into a small pocket and the overlaying skin closed. Sterile gauze was used as a primary covering over the wounds with Tegaderm™ as a secondary dressing. To protect the catheters and wounds, a custom-made swine jacket (Lomir Inc., Malone, NY, USA) was fitted to each animal. The swine recovered in their pen.

### 2.4. Blood Draw Analysis and Culturing

**Figure 1:**
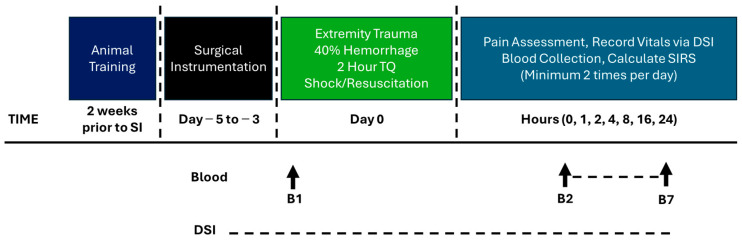
Model development Phase 1 timeline.

**Figure 2:**
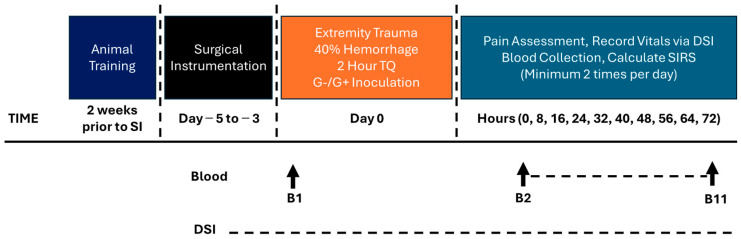
Model development Phase 2 timeline.

From conscious swine, blood samples were harvested pre- and post-injury, and at scheduled intervals (Figure 1 and Figure 2). Using standardized protocols (Siemens, ADVIA 2120i Hematology System with Multi Species Software Version 6.11.7), complete blood counts and chemistries were performed on each blood sample. The machine incorrectly calculated the differential on a few samples and is notated accordingly for the Lymphocyte and Neutrophil #’s. In addition, i-STAT^®^ was used for blood chemistry analysis, as previously described [21]. Aerobic blood culture bottles (BD BACTEC™, Franklin Lakes, NJ, USA) were inoculated with 10 mL of whole blood each and incubated at 35 °C in a (BD BACTEC™ FX40 instrument, Franklin Lakes, NJ, USA) for up to 5 days.

### 2.5. Establishment of SIRS Criteria for Sinclair Swine

Baseline values from all 26 animals were used to determine the Sinclair swine SIRS criteria. All animals underwent the same surgical instrumentation as stated above at least three days prior to injury. Telemetric data was processed in Ponemah V6.51 as previously described [21]. Briefly, data from the 48 h period prior to the morning of injury were included −56 to −8, two full 12 h cycles for each day and night period) for HR, RR, and temperature and were stratified by day/night cycles. The WBC counts were from blood collections from the initial screening and the same forty-eight-hour period to include the baseline blood collection from the morning of pre-injury. SIRS parameter cutoff criteria were determined using the mean values ± 2 standard deviations (SD), as previously performed [21,22], and incorporated the diurnal differences in RR, temperature, and HR. In alignment with accepted practice in the medical field, achievement of a positive SIRS state occurs once two or more SIRS parameters exceed the cutoff criteria [10,11,12,22].

### 2.6. Model Development Phase 1: Complex Extremity Trauma Injury (CETI)

On Day 0, animals were placed under anesthesia and received Lactated Ringers (LR) for fluid maintenance before initiation of extremity injury (ET) on the left leg using a combination of techniques.

A 5 cm × 3 cm × 8 cm trapezoidal skin excision was made 1 cm distal to the tibial tuberosity. Additionally, the fascia of the anterior and lateral compartments, as well as the periosteum, were excised [23]. Approximately 10 g of muscle from the anterior muscle compartment was removed with sharp dissection, and a 10 mm trephine was used to create a unicortical defect in the proximal tibia. Lastly, a thermal injury was performed on all muscle, fascia, periosteum, and bone within the wound. Specifically, a thermocoupled custom-made brass contact burn device [24], heated to 200 °C with a surface area of 25 cm^2^, was placed on the exposed tissue for 10 s. Any area of the wound not burned due to curvature was subjected to charring using electrocautery (Neptune^®^ SafeAir^®^ Smoke Evacuation Pencil, Stryker, Michigan, USA) as previously performed [23,25]. The burn was characterized by gross observation. The wound was then covered with gauze and Tegaderm™.

Following the ET, a 30–40% (*v*/*w*) estimated blood volume hemorrhage was performed over 45 min using a programmable pump (Masterflex DAQ system) at a rate of 1–2 mL/kg/min via the carotid catheter. Hemorrhage was paused if mean arterial blood pressure (MAP) dropped below 35 mmHg and was resumed above 40 mmHg. Initially, a shock period of approximately one hour was observed prior to administration of any resuscitation fluids. Shed blood was collected in 450 mL blood bags (TERUFLEX Blood Bag System with CPD/OPTISOL™ Solution, Termu Corporation, Tokyo, Japan). After the shock period, shed blood (up to 2 units, to mimic the amount a medic may have access to) was returned through the jugular vein catheter (1 mL/kg/min). To prevent hypocalcemia during hypovolemic shock, CaCl_2_ (1 g) was given IV in the same time frame as the resuscitation to replace the calcium bound by CPD in the shed blood. During the model development phase 1, the resuscitation volumes were reduced to induce a SIRS state.

A pneumatic tourniquet (TQ) (Model CMG-PTC, Delfi Medical Innovations, Inc., Vancouver, BC, Canada) was applied proximally to the knee for two hours with cuff inflation to 300 mmHg, occluding blood flow to the distal hind limb and injury site. To keep the TQ in place, the limb and TQ were fixed in a stabilization frame while the animal was in a dorsal recumbent position. For the rest of the manuscript, the term CETI will be used and refers to the Complex Extremity Trauma Injury which consists of the ET, hemorrhage, shock, resuscitation (if given), and TQ.

Following the procedure, the wound was wrapped in combinations of sterile gauze, Tegaderm™, non-stick elastic (e.g., VetWrap), and elastic adhesive tape (e.g., Elastikon). A jacket was reapplied after surgery. The swine regained consciousness from anesthesia then returned to their pen with food and water *ad libitum*. Twenty-four hours after CETI, surviving animals were anesthetized and euthanized. An intravenous overdose of pentobarbital was given (Fatal Plus, at least 150 mg/kg). The protocol timeline is depicted in Figure 1.

### 2.7. Model Development Phase 2: Bacterial Administration and Extended Monitoring

Phase 2 consisted of the same procedures as Phase 1 up until the TQ was deflated. At that point, the animals were moved to the animal biosafety level 2 portion of the vivarium for the inoculation procedures. The wound was inoculated with two multidrug resistant (MDR) bacteria, one Gram-positive (G+) and one Gram-negative (G−). The wound dressing was removed, and 1 mL each of (10^8^ CFU/mL in saline) bacterium was spread evenly over the wound surface with a cotton tipped applicator and left open for a five-minute period [23]. The G+ bacterium is *Staphylococcus aureus* (USA-300, JE2 [26]) and the G− bacteria are *Acinetobacter baumannii* (AB5075 [27]), *Klebsiella pneumoniae* (MRSN KP106625), and *Pseudomonas aeruginosa* (MRSN PA5519 [28]). The bacteria were prepared from frozen stock by thawing (37 °C) then conducting 3 washes with sterile saline (10 min, 4000× *g*, 4 °C). The bacteria were resuspended in sterile saline and kept on ice until inoculation (~30 min later). Following the procedure, the wound was wrapped in combinations of sterile gauze, Tegaderm™, non-stick elastic (e.g., VetWrap), and elastic adhesive tape (e.g., Elastikon). A jacket was reapplied after surgery. The swine regained consciousness from anesthesia then returned to their pen with food and water *ad libitum*. Seventy-two hours after CETI, surviving animals were anesthetized and euthanized. An intravenous overdose of pentobarbital was given (Fatal Plus, at least 150 mg/kg). The protocol timeline is depicted in Figure 2.

### 2.8. Tissue Plating

Tissue samples of the muscle were collected and weighed in sterile Stomacher bags (Seward BA6092) after euthanasia. For quantitative microbiology, 10 mL of sterile saline were added to each bag and placed in a Seward Stomacher 80 Lab System for 1 min at normal speed. A 48-well plate was used to make serial dilutions (10^0^–10^−6^) of each tissue sample and 100 µL of each dilution was plated on duplicate sets of blood agar plates (Remel™ Blood Agar, Monoplate, Lenexa, KS, USA). Plates were incubated for 18–24 h at 35–37 °C. Colony forming units were normalized to tissue weight.

### 2.9. Statistical Analysis

The sample size was guided by literature review and a power analysis. If the swine survived and recovered from the extremity trauma injury, they were included in all data analysis. Statistical evaluation was performed with GraphPad Prism 10.4 (GraphPad Software LLC, La Jolla, CA, USA). JMP 18 (JMP Statistical Discovery LLC, Cary, NC, USA) was used for outlier detection in all data, utilizing the Quantile Range Outlier Report tool; tail quantile = 0.25 and Q = 3. The Day to Night telemetry datasets failed a normality test; therefore, the nonparametric Mann–Whitney test to compare ranks was used. Mixed-effects analysis with the Dunnett multiple comparisons test was used for blood work and telemetry data. Mixed-effects analysis was required due to missing values from 2 animals not surviving until the 72 h endpoint. Continuous data are reported as the mean ± SD. For the SIRS status categorical dataset, the nonparametric Friedman test was used. The mean rank scores were compared to baseline values using Dunn’s method to correct for multiple comparisons. The median [interquartile range (IQR)] was used to report these datasets. Each parameter was compared to its baseline value, and the *p*-value was determined, with an α = 0.05 (statistical significance *p* < 0.05).

## 3. Results

### 3.1. Final SIRS Criteria for Sinclair Swine

**Figure 3:**
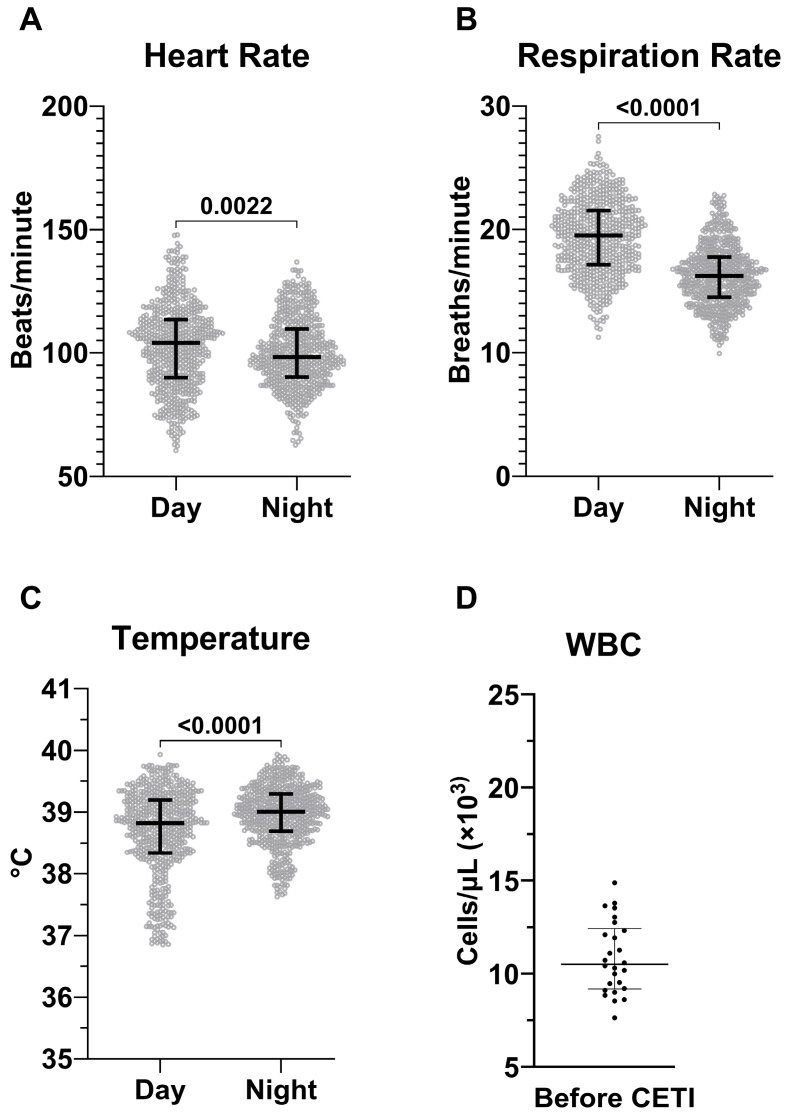
Determination of baseline values in Sinclair swine. Hourly averages of heart rate (**A**), respiration rate (**B**), and temperature (**C**) measured using telemetry. The data is acquired from N = 26 animals during a 48 h baseline period and is stratified by day and night cycles. White blood cell count (**D**) measured at blood draws prior to CETI of the same animals. HR, RR, and Temp are presented as median ± IQR while WBC is mean ± SD.

To our knowledge, SIRS criteria specific to adult male Sinclair swine have not been determined. Therefore, data from 26 adult male Sinclair swine was used to establish baseline values relevant to SIRS criteria (HR, RR, temperature, WBC) for this species (Figure 3). HR, RR, and temperature each showed significant diurnal variation, with significant differences between day and night periods. These fluctuations were considered in the final analysis and form the foundation for applying SIRS criteria to the subsequent model development (Table 1).

### 3.2. Model Development Phase 1: CETI

**Figure 4:**
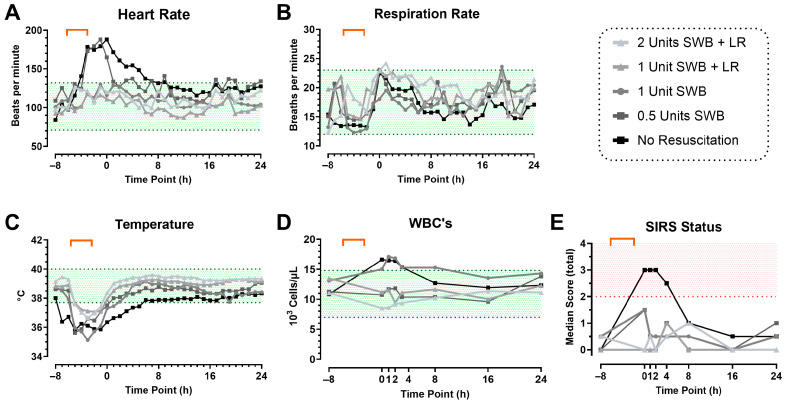
The role of resuscitation on SIRS status during model development phase 1. SIRS criteria: heart rate (**A**), respiration rate (**B**), temperature (**C**), and WBC count (**D**) up to 24 h after CETI. The shaded green area indicates the identified normal range of values in Sinclair swine (Table 1). Calculated SIRS scores (**E**), with positive SIRS status (≥2) indicated with red shading. Color gradient represents reduced resuscitation (from gray to black). Orange bracket indicates approximate timeframe of CETI. SWB = shed whole blood; LR = Lactated Ringer’s solution. N = 11 animals.

In Phase 1, eleven swine were subjected to CETI as depicted in Figure 1. In this phase, we evaluated if reducing resuscitation would induce a SIRS state (Figure 4). A pronounced SIRS response was observed in animals for which shed whole blood and/or fluid resuscitation were withheld. Shortly after injury, these swine exhibited values outside the established normal ranges (Table 1) for HR (188 ± 1 bpm), temperature (36.36 ± 0.89 °C), and WBC (16.61 ± 1.10 × 10^3^/µL). These abnormalities persisted for at least four hours after trauma before gradually normalizing by the eight-hour mark, though they remained elevated compared to their starting values. Overall, SIRS assessment showed that animals in the no-resuscitation group consistently achieved a positive SIRS diagnosis, with a score of ≥2 based on the defined criteria, and remained in a SIRS state for the first 4 h after CETI (Figure 4E).

### 3.3. Model Development Phase 2: Inoculation with Bacteria and Prolonged Monitoring

After successfully inducing SIRS from severe extremity trauma over a 24 h observation period, the protocol duration was extended to 72 h. Two animals were monitored constantly after injury induction, demonstrating the survivability of this model out to 72 h.

**Figure 5:**
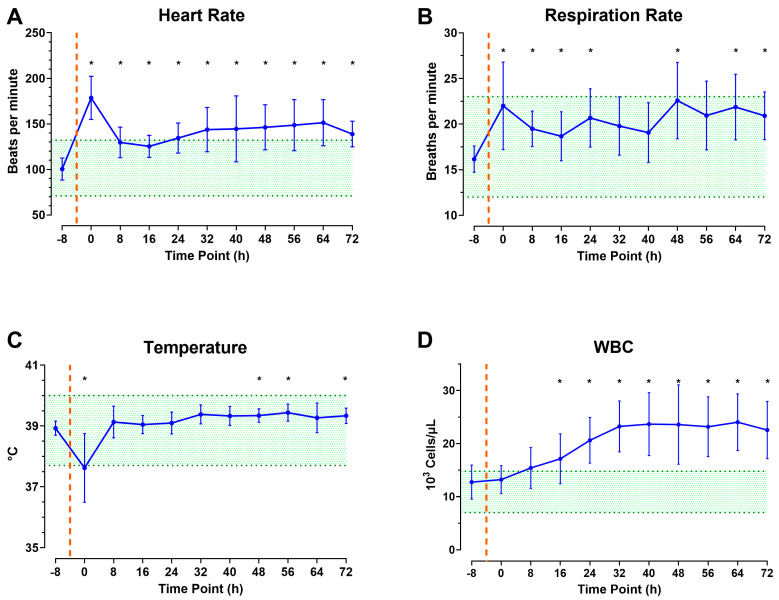
The role of infection and prolonged monitoring on SIRS status during model development phase 2: Heart rate (**A**), respiration rate (**B**), temperature (**C**), and white blood cell count (**D**) up to 72 h after CETI. Orange dashed line signifies estimated time of injury and green shading represents normal values. N = 9 animals. Data are presented as mean ± SD; * *p* < 0.05 compared to baseline.

A local bacterial infection with various G+ and G− bacteria was induced following CETI as depicted in Figure 2. Nine animals were used to refine the model by adding pathogens to the wound. When pathogens were introduced to the wound, the HR immediately peaked and was significantly elevated compared to baseline for the duration of the experiment (Figure 5A). RR and temperature remained within the normal range (Figure 5B,C). However, RR remained in the upper range of normal values throughout the study, with significantly increased values compared to baseline at all timepoints except hours 32, 40, and 56. Temperature dropped immediately, then recovered, and was significantly increased above baseline values at 48, 56, and 72 h. WBC counts gradually increased over the 72 h observation period and remained significantly elevated compared to baseline from the 16 h timepoint until the end (Figure 5D).

**Figure 6:**
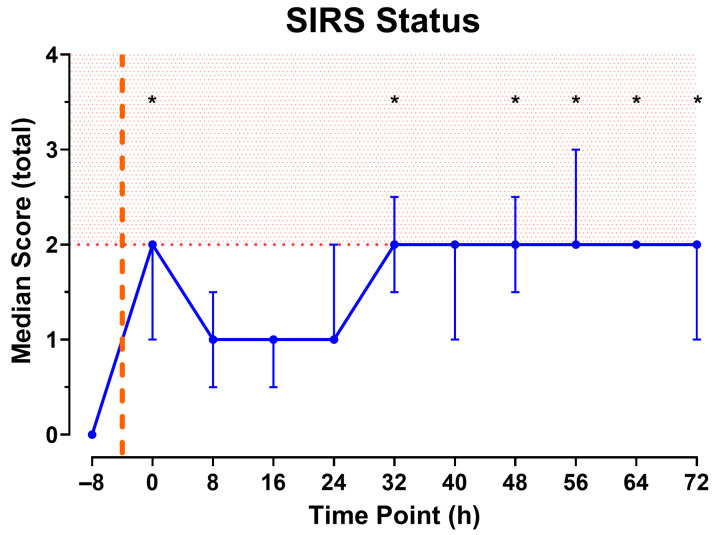
Total SIRS Score calculated from measurements collected every 8 h. Red shading indicates SIRS state. Orange dashed line represents approximate time of injury. N = 9 animals. Data shown as median [IQR]. * Indicates fulfillment of SIRS criteria and *p* < 0.05 significance compared to baseline (−8).

As shown in Figure 6, combining the data of the individual parameters reveals that the total SIRS score meets the criteria of two or more immediately after injury, then declines until the 32 h observation period. At this point, the minimum score of two is sustained until euthanasia at the 72 h end point.

### 3.4. Local Wound Infection

Immediately after the injury, the wound presented with burns, charred tissue, and exposed bone, as well as a deep, circular bone defect and diffuse bleeding sites (Supplemental Material Figure S1). Red, vital muscle tissue was present, and the wound edges were smooth. There were no signs of infection. After 72 h the wound bed exhibited green and yellow purulent exudate, avital, grayish muscular tissue without signs of adequate perfusion during gross wound inspection. Additionally, the wound edges were uneven and edematous with purulent components. The surrounding tissue was edematous and there was a foul odor.

Overall, the burden of both G+ and G− bacteria were observed with 3.32 × 10^6^ CFU/g [1.96 × 10^6^, 8.43 × 10^6^] and 7.4 × 10^5^ CFU/g [9.78 × 10^4^, 5.67 × 10^6^]. Additionally, blood bottles were negative for bacterial growth.

### 3.5. Blood Biochemistry Laboratory and Organ Dysfunction

Analysis of the blood chemistry data from all inoculated animals revealed significant but transient changes compared to baseline values related to different organ systems (Table 2). Several parameters had significantly elevated values immediately after injury: lactate (7.2 ± 4.0 mmol/L), creatinine (1.87 ± 0.41 mg/dL), BUN (19.3 ± 3.2 mg/dL), and glucose (181 ± 43 mg/dL). After 24 h, AST (248 ± 213 U/L) and BUN (13.9 ± 4.3 mg/dL) were significantly elevated.

Changes in cellular blood components were also observed during the 72 h observation period. There was a substantial decrease in platelet levels within the initial 24 h period, followed by a notable increase at the 72 h mark. An immediate and sustained significant decrease was observed in red blood cells (RBCs), hematocrit (HCT), and hemoglobin (Hb). The lowest HCT value (23.20 ± 3.66%) was recorded after 48 h, as was the lowest Hb value (7.53 ± 0.95 g/dL). Following injury, there was a substantial decrease in lymphocyte concentration (2.93 ± 0.53 × 10^3^ cells/µL). However, the neutrophil count remained significantly higher after injury and bacterial inoculation, peaking at 48 h post-injury at 14.38 ± 5.55 × 10^3^ cells/µL.

**Table 2:** Laboratory data from bacteria-inoculated animals.

Analyte	Baseline	0 h	24 h	48 h	72 h
Lactate (mM)	1.4 ± 0.4	7.2 ± 4.0 *	2.8 ± 2.5	2.6 ± 2.5	2.1 ± 1.5
Glucose (mg/dL)	97 ± 8	181 ± 43 *	112 ± 17	101 ± 22	100 ± 20
ALT (U/L)	57 ± 8	56 ± 12	96 ± 49	97 ± 52	89 ± 50
AST (U/L)	41 ± 20	159 ± 157	248 ± 213	139 ± 121	66 ± 59
ALP (IU/L)	61 ± 14	62 ± 13	65 ± 16	74 ± 79	71 ± 96
GGT (U/L)	42 ± 8	40 ± 6	39 ± 6	40 ± 5	41 ± 6
Albumin (mM)	1.1 ± 0.1	1.0 ± 0.1 *	1.0 ± 0.1 *	0.9 ± 0.1 *	0.9 ± 0.1 *
INR	1.25 ± 0.20	1.27 ± 0.16	1.32 ± 0.13	1.29 ± 0.09	1.81 ± 1.61
Fibrinogen (mg/dL)	347 ± 67	250 ± 65 *	513 ± 58 *	622 ± 64 *	669 ± 88 *
D-Dimer (µg/mL)	0.24 ± 0.11	0.22 ± 0.11	0.17 ± 0.08	0.22 ± 0.09	0.19 ± 0.09
PT (sec)	16.1 ± 2.0	16.3 ± 1.6	16.9 ± 1.3	16.6 ± 1.0	20.6 ± 12.4
a PTT (sec)	57.1 ± 24.2	43.8 ± 17.5	77.3 ± 38.5	57.2 ± 32.8	65.6 ± 70.8
BUN (mg/dL)	7.7 ± 2.2	19.3 ± 3.2 *	13.9 ± 4.3 *	9.8 ± 6.9	10.0 ± 9.0
Creatinine (mg/dL)	0.95 ± 0.13	1.87 ± 0.41 *	0.99 ± 0.18	0.95 ± 0.30	0.96 ± 0.32
Lymphocytes (10^3^ cells/µL)	5.82 ± 0.77 ^1^	2.93 ± 0.53 *^,1^	7.09 ± 2.85 ^2^	6.53 ± 2.73 ^1^	5.96 ± 2.73 ^1^
Neutrophils (10^3^ cells/µL)	5.37 ± 2.53 ^1^	9.57 ± 2.66 *^,1^	9.71 ± 6.60 ^2^	14.38 ± 5.55 *^,1^	13.10 ± 5.61 *^,1^
Platelets (10^3^ cells/µL)	356 ± 70	240 ± 46 *	276 ± 61 *	320 ± 127	500 ± 164 *
Red Blood Cells (10^6^ cells/µL)	5.52 ± 0.36 ^1^	5.24 ± 0.58 ^1^	3.87 ± 0.43 *	3.61 ± 0.37 *	3.71 ± 0.43 *
Hemoglobin (g/dL)	11.98 ± 1.04	11.46 ± 1.37	8.46 ± 1.36 *	7.53 ± 0.95 *	7.71 ± 1.05 *
Hematocrit (%)	35.84 ± 3.52	34.40 ± 4.68	25.57 ± 4.32 *	23.20 ± 3.66 *	23.96 ± 3.64 *

Data are presented as mean ± SD, * *p* < 0.05 compared to baseline; N = 9 animals. ^1^ N = 8; ^2^ N = 7.

## 4. Discussion

We have successfully developed a deliberate, complex extremity trauma model that specifically takes into account the wound characteristics typical of severe extremity injuries, such as those that occur in combat injuries. These wounds often include disruption of the skin barrier, soft tissue and bone damage, muscle loss, and resulting hemorrhage. Additionally, thermal injuries can characterize these wounds, especially when caused by blasts [29,30]. Therefore, it was imperative to include these injury types when developing a model of inducing a SIRS state from extremity trauma. Although the application of a TQ in severe hemorrhage can be life-saving, its use is not benign, as it creates ischemia distal to TQ placement, and thus can cause reperfusion injury if not removed in an appropriate time [31,32]. To mimic this standard component of acute extremity injury involving hemorrhage and add the distinct injury pattern induced by torniquet-induced limb ischemia–reperfusion injury, a temporary 2 h TQ application was included. Initially, the resuscitation volume included up to two units of shed whole blood and lactated Ringer’s solution. However, it was ultimately reduced and eliminated during model development phase 1. This resulted in the development of a SIRS state, defined by the refined SIRS criteria for Sinclair swine, within the first four hours after injury, illustrating the impact of injury and shock on the body (Figure 4). The animals exhibited elevated heart and respiratory rates in response to severe tissue damage and blood loss. This reaction was likely intensified by the release of endogenous damage-associated molecular patterns (DAMPs), which triggered a cascade of sterile inflammation. This resulting uncontrolled immune response, driven by the interaction of the hemostatic, inflammatory, endocrine, and neurological systems, ultimately led to further tissue damage [33,34,35,36,37].

After successfully completing phase 1, the protocol duration was prolonged to 72 h to mimic delayed evacuation. Both of the two animals used survived until the end of the study. This makes our model stand out from existing models, which mostly study sedated animals over shorter periods of time [38].

After confirming survival to 72 h, a local bacterial infection involving both G+ and G− bacteria was induced following the CETI to simulate bacterial contamination in severe limb injuries, and introduce the other etiological pathway of SIRS into the model, by activating immunological responses through pathogen-associated molecular patterns (PAMPs) [11,35]. This potentially exacerbates the inflammatory response to trauma and increases the relevance of this model for clinical situations, such as those seen in severe limb injuries in military settings. This approach is based on the potential for bone infection in open fractures, which create additional entry points for bacteria and activate the body’s defense mechanisms, which is reflected by the elevated leukocyte and neutrophil counts we observed (Figure 4). After 72 h, it was demonstrated that, while the body could contain the tissue infection locally, it could not eliminate it, as the recovered bacterial quantity of the muscle remained above the 10^5^ CFU/g threshold for both G+ and G− bacteria.

SIRS is characterized by increased HR, temperature, RR, and WBC [10,12] with at least two parameters exceeding certain thresholds required to define a SIRS state. To apply the clinically established SIRS criteria scoring system to our Sinclair pig model, we have adjusted the cutoff values using our baseline data that represents normal, healthy, uninjured animals (Table 1). This adjustment accounts for the physiological and anatomical differences between swine and humans, thereby enhancing the model’s translatability to human conditions [39]. Applying our developed SIRS criteria to this new extremity trauma model, we effectively demonstrate the progression of SIRS as observed in severe extremity wounds, illustrating that the combination of injury and contamination can affect the entire organism.

During model development phase 1, HR exhibited robust changes when resuscitation support was reduced or eliminated; a trait preserved in phase 2. Furthermore, the tachycardia phase immediately after CETI was accompanied by a return to normal HR values in all animals within the eight- to sixteen-hour period, but with a notable rise over the remainder of the study period(s) and disruption of circadian rhythm. Similarly, this was also seen in a multiple trauma model with severe hemorrhage of 40% blood volume, with an increased HR of up to 170 ± 39 bpm, which decreased but remained elevated at 84 ± 23 bpm until the 72 h study endpoint [40]. This phenomenon may be explained by the spleen’s auto-resuscitative function in swine, leading to erythrocyte mobilization and thus increasing circulating blood volume and enhancing arterial oxygen carrying capacity [41]. Additionally, this steady HR climb has been identified in other studies of SIRS and sepsis in swine. Soerensen et al. demonstrated a steady survivable increase in HR over a 48 h period in a model of IV-infused G+ pig sepsis [42]. Nguyen et al. also described significant tachycardia over a 70 h period after bacteremia induction [43]. Thus, the increase in HR may be attributable to the organism’s compensation mechanisms of hemorrhagic shock aggravated by the response to inoculated bacteria [44,45].

The RR increased in all animals after the procedure and during the shock period compared to the baseline value at the −8 h pre-CETI timepoint. However, according to our SIRS criteria, the animals did not fulfill the criteria, despite having a higher RR than the baseline value. Notably, infected animals demonstrated a steady but elevated RR throughout the observation period. An increase in RR is often the earliest indication of SIRS [46].

Temperature decreased and subsequently increased compared to defined normal values. The initial blood loss and the resulting shock state can contribute to hypothermia by the impairment of thermoregulation [47,48]. Additionally, anesthetic medication during the surgical procedure of CETI may have influenced thermoregulation, causing a reduction in body temperature [49]. Similarly to RR, diurnal temperature fluctuation appeared to be absent in infected animals. Their body temperatures remained above the determined mean temperature values (38.53 °C, daytime and 38.91 °C, nighttime) and within the upper normal range until the end of the observation period. This may be indicative of an impaired thermoregulatory response during inflammation and infection [50].

In phase 1, an overall significant increase in WBC count was observed, which can be attributed to reperfusion following two hours of TQ application. TQ fundamentals rely on blood and fluid occlusion, which leads to acute ischemia. The release of the TQ can lead to reperfusion injury, possibly leading to the release of active inflammatory mediators from local sites. The systemic inflammatory reaction can be further intensified by the subsequent release of additional inflammatory cells from the liver and gastrointestinal tract [51,52]. In phase 2, the infected animals’ WBC counts significantly increased, surpassing the normal range. This emphasizes the enhanced inflammatory reaction to bacterial application [53].

Blood sample analysis revealed systemic changes following severe trauma and infection. Our findings indicate transient organ impairment, particularly renal dysfunction, as evidenced by elevated creatinine and BUN levels. The literature attributes the prevalence of acute kidney injury in severe trauma to multiple factors, including the severity of the injury, hemorrhagic shock with restricted renal perfusion, rhabdomyolysis, and traumatic inflammation, all of which can lead to reduced kidney function [51,54,55,56]. Liver stress was indicated by elevated levels of AST and total bilirubin in our model. Liver dysfunction can also be associated with systemic ischemia [57]. Additionally, in the acute phase of SIRS, similar increases are consistent with SIRS induced by burns [22]. Our results are supported by Cralley et al., who monitored similar changes in creatinine and bilirubin levels during severe shock over their four-hour observation period [58]. Nguyen et al. developed a bacterial infection model illustrating how infection affects the kidneys and liver [43]. After 72 h, macroscopically visible changes in these organs were detected. These findings provide further evidence of the pathogenic impact of bacterial infections and may explain the pathological changes in our model’s values.

As anticipated, following a hemorrhage without resuscitation, reduced hematocrit and hemoglobin levels indicated anemia. Together with decreased blood pressure and elevated HR, indicated as positive shock index, these findings confirm the severity of the induced hypovolemic shock [59,60]. Furthermore, the detected hyperlactatemia reflects impaired tissue oxygenation and metabolic stress [51,61]. The tendency of values towards the baseline outlines the power of the auto-resuscitative properties of the spleen, as described above.

Investigated animals showed no significant coagulation impairment, differing from end stages of severe SIRS in humans [11,62]. However, changes in fibrinogen concentration were detected, with elevated concentrations from 24 h to the end of the study. There were also changes in platelet concentration from 24 h to the end of study: a significant reduction within the first 24 h, followed by an increase of up to 517 ± 152 × 10^3^ cells/µL at 72 h.

This study allowed us to develop a reproducible conscious animal model of systemic inflammatory response syndrome that considers the multifaceted characteristics of severe extremity trauma. This provides a valuable foundation for future combat medicine research into diagnostics, the testing of point-of-care devices, and new clinical decision support tools and therapeutic approaches. Using adapted SIRS criteria enables this preclinical animal research to be translated into human conditions. Furthermore, this model may be further used to investigate sepsis after identification of the correct inoculation procedures.

### Limitations of This Study

Several limitations are inherent in this study. Adult male Sinclair miniature swine were selected due to their anatomical and physiological similarities to humans and male-centric roles in combat. To maintain consistency and reduce biological variability, only male swine were used in this study. This limits the applicability of the results to a broader patient population composed of both males and females. In addition, diagnostic capabilities in swine are inherently limited [18,19]. Despite this, the use of the implanted DSI telemetry device facilitated continuous monitoring of the awake animals, thereby improving assessment and replicating information gathered from awake patients. Unfortunately, the study design did not allow for comprehensive tracking of urine output and fluid balance in awake animals; instead, renal function was monitored through blood sample analysis.

As described above, the presence of the spleen may have influenced hemodynamic variability and enhanced recovery after hemorrhagic shock. However, due to the spleen’s central role in the immune system, we refrained from performing a splenectomy in order to preserve an innate immune response. Furthermore, the spleen is thought to store up to a unit of blood, and as we observed significantly decreasing values for RBCs, hematocrit, and hemoglobin, it could be argued that a splenectomy may have worsened these values, and a model surviving to 72 h may not have been possible.

Furthermore, this study did not include cytokine analyses, reflecting the intention to develop a model that emphasizes observable physiological changes rather than relying on complex, often unavailable, time-consuming laboratory inflammatory assays. While this enhances the model’s practicality in real-world conditions, it limits our understanding of immune signaling and cellular response patterns. Future studies are planned to consider cytokines in the investigation, in addition to expecting to be able to detect early changes in coagulation. The use of young, healthy swine also has limitations, as these animals lack the comorbidities and age-related immune changes often present in human patients. However, the military population predominantly consists of young and healthy service members, closely aligning with the original healthy state of the swine.

Additionally, the injury pattern employed in this study is representative of targeted extremity trauma. In contrast, real-life military or civilian trauma scenarios often involve multiple injuries, such as additional traumatic brain injury and thoracic or abdominal trauma. These combined injuries exacerbate systemic inflammation, promoting a faster and more severe progression of SIRS and multiple organ dysfunction. The discrepancy between experimental and clinical trauma scenarios highlights the limitations of applying this model’s findings to more complex clinical contexts. Nevertheless, this specifically allows for the investigation of the individual effects of complex extremity trauma on the entire organism. Furthermore, our study involved inducing a controlled bacterial infection with a specific combination of pathogens. While this approach ensures reproducibility, it does not fully reflect the complex nature of real-world infections, particularly in military trauma situations where wounds may be contaminated by various environmental bacteria, foreign materials, and resistant organisms. The complexity of this microbial flora could influence the course and severity of SIRS, which may not be adequately captured in this simplified model. However, in future studies, bacteria strains can be easily exchanged and added to the model as needed.

Moreover, the duration of the TQ application had to be determined. Our literature search revealed that evacuation times in past deployments ranged around 90 min [63]. In addition, two hours are defined as the critical threshold for the possible replacement or conversion of a TQ [64]. Therefore, a two-hour ischemic time was selected to reflect the upper limit of safe application. However, in the expected large-scale operations of the future, prolonged care and TQ times may be even longer. This issue will need to be addressed separately in future research using this model.

Lastly, independent external validation of the study’s results would have strengthened the robustness of this model, and this will be incorporated in ongoing aims of the study.

## 5. Conclusions

This is the first study to develop a severe extremity injury model that meets SIRS criteria for Sinclair swine. Our study lays the groundwork for developing a porcine model that mimics the systemic inflammatory response following severe limb trauma. This model will facilitate future research in prevention, diagnostics, and treatment by enabling precise monitoring of the animal’s condition throughout a 72 h time period, including real-time data on vitals and pathophysiology.

## Figures and Tables

**Table 1:** Sinclair swine SIRS Criteria.

Parameter	Time of Day	Average	SD	SIRS (+2 SD)	SIRS (−2 SD)
HR [bpm]	Day	103	17	137	69
	Night	100	14	128	72
RR [brpm]	Day	19	3	25	13
	Night	16	2.5	21	11
Temp [°C]	Day	38.70	0.70	40.10	37.31
	Night	39.00	0.47	39.94	38.06
WBC [10^3^/µL]		10.90	1.93	14.76	7.04

## Data Availability

Due to the size of the raw files, datasets are available upon request.

## Supplementary Materials

The following supporting information can be downloaded at: https://www.mdpi.com/article/10.3390/pathophysiology32040059/s1, Figure S1: Representative Digital Images of CETI Site.

## Abbreviations

The following abbreviations are used in this manuscript:

ALT	Alanine Transaminase
ALP	Alkaline Phosphatase
AST	Aspartate Transaminase
BP	Blood Pressure
bpm	Beats per Minute
brpm	Breaths per Minute
BUN	Blood Urea Nitrogen
CETI	Complex Extremity Trauma Injury
CFU	Colony Forming Unit
DSI	Data Sciences International
GGT	Gamma-glutamyl Transferase
Hb	Hemoglobin
HCT	Hematocrit
HR	Heart Rate
IM	Intramuscular
INR	International Normalized Ratio
IQR	Interquartile Range
LR	Lactated Ringer’s Solution
MAP	Mean Arterial Pressure
MDR	Multidrug resistant
RBCs	Red Blood Cells
RR	Respiration Rate
SIRS	Systemic Inflammatory Response Syndrome
SD	Standard Deviation
SWB	Shed Whole Blood
Temp	Temperature
TQ	Tourniquet
WBC	White Blood Cell
