# Supplementary material for: Polymicrobial Infection (Gram-Positive and Gram-Negative) Exacerbates Systemic Inflammatory Response Syndrome in a Conscious Swine Extremity Trauma Model
Source: Pathophysiology. 2025 Nov 4;32(4):59. doi: 10.3390/pathophysiology32040059 (PMC12641754; doi:10.3390/pathophysiology32040059)
Supplement: Supplementary file 1 [file pathophysiology-32-00059-s001.zip › pathophysiology-3872202-supplementary.pdf]

Supplementary Material

Figure S1 Representative Digital Images of CETI Site.

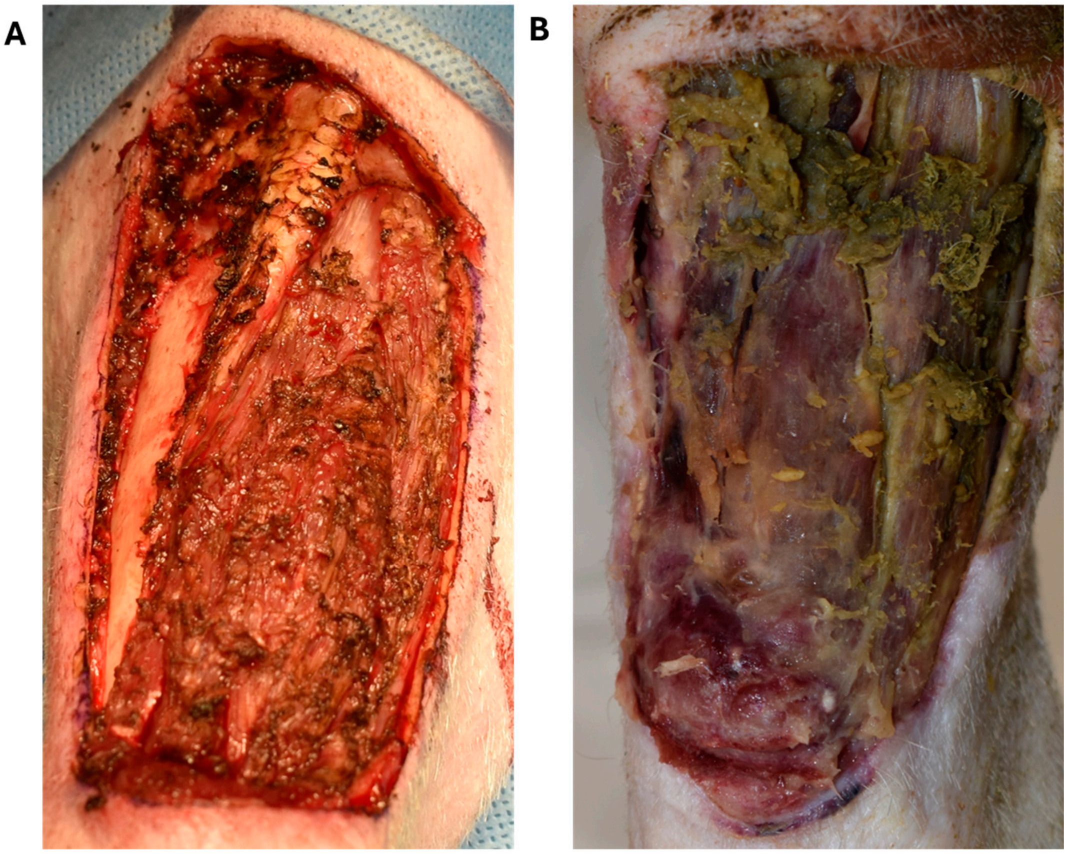

Comparison of wounded tissue immediately after CETI (A) and after the 72-hour observation time (B).
